# Supplementary material for: Cloud BioLinux: pre-configured and on-demand bioinformatics computing for the genomics community
Source: BMC Bioinformatics. 2012 Mar 19;13:42. doi: 10.1186/1471-2105-13-42 (PMC3372431; doi:10.1186/1471-2105-13-42)
Supplement: Additional file 1 — Supplementary 1 Cloud BioLinux software documentation in the form of a mini, self-contained website. Users need to download and uncompress the .zip file, and open through a web browser the "index.html" file available on the main directory. (ZIP 1823 kb). [file 1471-2105-13-42-S1.ZIP › Cloud-BioLinux-Package-Documentation/docs/BiodiversityR.html]

Bio-Linux Software Documentation Pages

Back to search form

## BiodiversityR

|  |  |
| --- | --- |
| Name | BiodiversityR |
| Description | BiodiversityR is an R library that provides an extra menu in the R Commander graphical interface for R.  To run R Commander with the BiodiversityR menu, start up R on your Bio-Linux system by typing `R` on the command line. Then within R, type: `library(BiodiversityR)  BiodiversityRGUI()`     Please note that the commands above are case-specific, and you must include the brackets written above.  You can obtain documentation about BiodiversityR from the "Help about BiodiversityR" option under the BiodiversityR menu in Rcmdr. |
| Homepage | http://cran.r-project.org/web/packages/BiodiversityR/index.html |
| Remote Documentation |  |
